# Online NIR Analysis and Prediction Model for Synthesis Process of Ethyl 2-Chloropropionate

**DOI:** 10.1155/2015/145315

**Published:** 2015-08-20

**Authors:** Wei Zhang, Hang Song, Jing Lu, Wen Liu, Lirong Nie, Shun Yao

**Affiliations:** School of Chemical Engineering, Sichuan University, Chengdu 610065, China

## Abstract

Online near-infrared spectroscopy was used as a process analysis technique in the synthesis of 2-chloropropionate for the first time. Then, the partial least squares regression (PLSR) quantitative model of the product solution concentration was established and optimized. Correlation coefficient (*R* ^2^) of partial least squares regression (PLSR) calibration model was 0.9944, and the root mean square error of correction (RMSEC) was 0.018105 mol/L. These values of PLSR and RMSEC could prove that the quantitative calibration model had good performance. Moreover, the root mean square error of prediction (RMSEP) of validation set was 0.036429 mol/L. The results were very similar to those of offline gas chromatographic analysis, which could prove the method was valid.

## 1. Introduction

Ethyl 2-chloropropionate (CAS number 535-13-7) is a clear colorless liquid with a pungent odor. Its flash point is 100°F and it is denser than water and insoluble in water. In recent years, as an important chemical intermediate and industrial reagent, it has been popularly applied in the synthesis of herbicides (e.g., phenoxypropionates, 2-(4-hydroxyphenoxy)propionate, and amino (or aryloxy) sulfonyl phenoxy propanates), plant auxiliaries (e.g., 2-chloroethyl trimethyl ammonium chloride, dimethylaminosuccinic acid), nonsteroidal antipyretic and anti-inflammatory drugs (e.g., naproxen, indomethacin, and ibuprofen), and so forth. Though the synthetic process of ethyl 2-chloropropionate is relatively simple, the current offline quantitative method for its production monitoring and quality control can hardly meet the requirements of related researchers and producers.

Previously, Food and Drug Administration (FDA) issued a guidance document to pharmaceutical industry regarding the implementation of process analytical technology (PAT) in 2004. Process analytical technology (PAT) has been defined as “a system for designing, analyzing, and controlling manufacturing through timely measurements of critical quality and performance attributes of raw and in-process materials and processes, with the goal of ensuring final product quality” [1]. Recently, the application of near-infrared (NIR) spectroscopy has grown rapidly as an efficient online monitoring technique [2], which has been used as an ideal tool for PAT. The growing concentration on NIR is probably a direct result for its advantages of outstanding sensitivity, high speed, low noise, nondestruction, and enabling the analysis of complex samples without the need for pure samples compared to others [3–5]. Near-infrared (NIR) spectroscopy was used as a process analytical technology to monitor the amino acids concentration profile during hydrolysis process of* Cornu Bubali* by Wu et al. [6]. And the use of near-infrared diffuse reflectance spectroscopy for qualification of* Ginkgo biloba* extract was described as raw material for use in pharmaceutical products by Rose and coworkers [7]. NIR spectroscopy has also been used as an analyzer to determine the effect of several operating conditions on recovery, selectivity, and productivity for production of methyl isobutyl ketone (MIBK). The use of this PAT approach enabled the researchers to perform the necessary experiments in a time-efficient fashion and resulted in 30% improved productivity of MIBK [8].

Based on the above research status, the aim of this study was to use UV-NIR spectroscopy for online and nondestructive analysis of synthesis process of 2-chloropropionate catalyzed by ion exchange resin for the first time. The method of model updating was utilized to make the models more accurate and obtained better prediction results. The concentration values were very close to those obtained by offline gas chromatographic analysis. The developed method was supposed to provide foundation for further process chemical analysis and useful reference for similar online analytical research of synthetic reaction.

## 2. Experimental

### 2.1. Chemicals and Materials

All reagents used were of analytical grade. Methanol, ethanol, 2-chloropropionic acid (purity: 0.9956%), and ethyl 2-chloropropionate (purity: 0.9913%) were obtained from Kelong Chemical Inc. (Chengdu, China). D001 strong acidic cation exchange resin was purchased from Shengquan Chemical Inc. (Langfang, China). NIR spectrometer (NIRQUEST512), DH-2000-BAL deuterium light source, and optical fiber (T300-UV-VIS) were obtained from Ocean Optics Inc. (Dunedin, USA). Norprene chemical tubing was purchased from Saint Gobain Inc. (USA). GC-7900 gas chromatographic system (Tianmei Scientific Instrumental Inc., Shanghai, China) was used in the quantitative analysis, which was equipped with GH-300 hydrogen generator and flame ionization detector.

### 2.2. Experimental Procedure and Equipment

**Figure 1:**
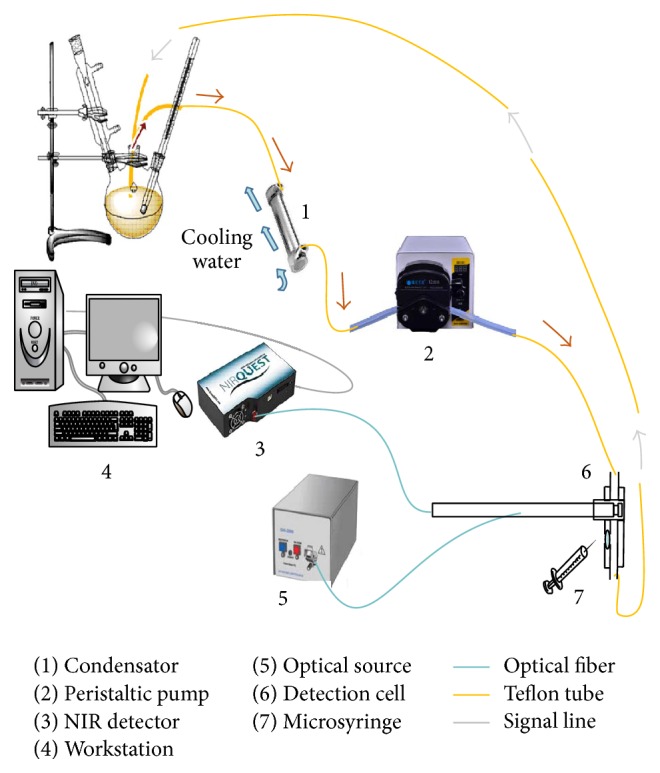
Experimental facility.

The scheme of experimental device is shown in Figure 1, which includes condensator, peristaltic pump, NIR detector, workstation, optical source, detection cell, microsyringe connected with teflon tube, optical fiber, and signal line. In the three-necked flask, 2-chloropropionic acid was esterified with ethanol under the catalysis of strong acidic cation exchange resin. Because the synthesis of ethyl 2-chloropropionate was performed at 110°C under refluxing, the condensator was added before the NIR spectrometer in order to eliminate effects of temperature fluctuation on spectral signals. Meanwhile, the peristaltic pump was adjusted with the stable flow rate of 0.2 mL/s through repeated experiments to meet the requirement of ideal online detection. In the self-made detection cell, the fiber-optic probe acquired useful NIR signals and the microsyringe could collect the samples in the sampling port. Then, the sample solution was analyzed with GC method. The concentration (*C*) of the product ethyl 2-chloropropionate can be obtained through the following equation:

(1) $$\begin{array}{c} C=\frac{C_{0}A_{2}}{\left(A_{2}+fA_{1}\right)}, \end{array}$$

where *C*_0_ is the initial concentration of 2-chloropropionic acid ethanol solution, *A*_1_ and *A*_2_ are the peak areas of 2-chloropropionic acid and ethyl 2-chloropropionate in the collected samples, respectively, and *f* is the relative correction factor of 2-chloropropionic acid to ethyl 2-chloropropionate.

### 2.3. Gas Chromatographic Analysis

**Figure 2:**
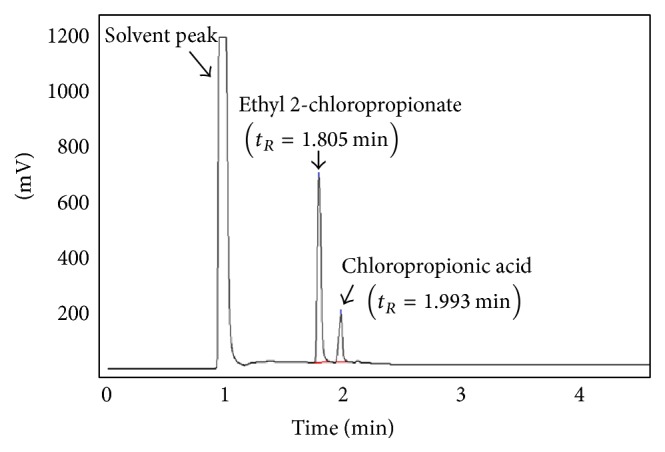
GC chromatogram of ethanol, ethyl 2-chloropropionate, and 2-chloropropionic acid.

The sample of reaction product was drawn out from the pipeline of PAT system at regular intervals. It was diluted 5 times with dehydrated alcohol and then was filtrated by 0.22 m Millipore filter. 5 *μ*L sample solution was injected into GC instrument and analyzed under the following conditions: nonpolar TM-1 capillary column (15 m × 0.53 × 0.5 *μ*m), temperature programming (started at 90°C, holding for 0.5 min, and then increased to 165°C at a rate of 25°C/min), the temperature of injection port, and FID detector under 180°C, N_2_ at 65 mL/min as carrier gas. As a result, the solvent of alcohol, the product of ethyl 2-chloropropionate (*t*_*R*_ = 1.805 min), and the substrate of 2-chloropropionic acid (*t*_*R*_ = 1.993 min) could reach the baseline separation in 3 minutes. The results of GC chromatogram of ethanol, ethyl 2-chloropropionate, and 2-chloropropionic acid are shown in Figure 2.

### 2.4. NIR Spectroscopy Collection

The NIR spectroscopic data of training set for the reaction process were collected with 3 replications continuously every 30 s, and the average value was determined as the spectral absorption data of the sample at this time. Data of calibration set were the average of spectral values in three times which were collected once every 1 min in another batch synthetic process [9].

**Figure 3:**
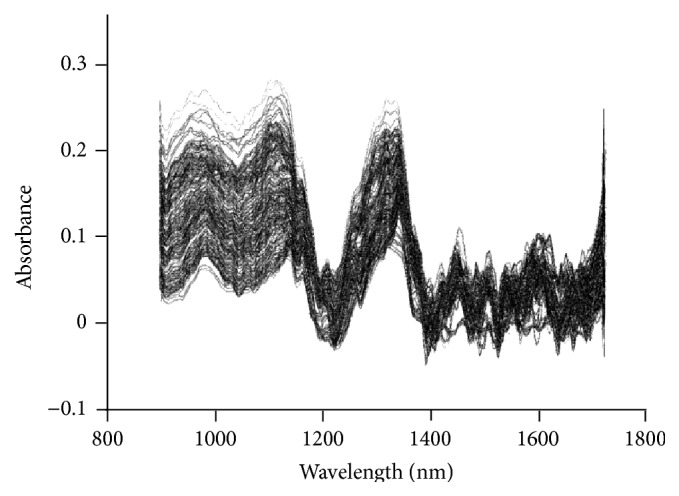
The stacked NIR spectra of all the samples.

Spectra acquisition conditions were presented as follows: in the transmission mode, the background of unreacted solution was used as a reference, and the detection wavelength ranged from 900 nm to 1800 nm with a resolution of 4 nm; an integral time was 280 ms and the optical path was 2 cm. The stacked original NIR spectra of all the samples are shown in Figure 3.

### 2.5. Data Analysis

Spectral data were manipulated by identifying the optimal spectral regions and selecting appropriate pretreatment methods, and then they were correlated with the data measured by the reference assays using PLSR to develop calibration models [10, 11]. The performance of the calibration models was assessed in terms of root mean square error (RMSE), correlation coefficient (*R* ^2^), root mean square error of cross-validation (RMSECV), and relative standard error of prediction (RSEP) [12].

In PLSR algorithm, including more PLSR factors in the model would better fit the modeling data, but the prediction accuracy of the other samples might become worse. This phenomenon was called “overfitting” of a model. In this case, the corresponding components should be eliminated effectively (including noise, nonspectral measurement information) [13, 14]. The calibration models with the highest *R* and the lowest RMSEC and RMSEP with the least difference from each other were considered optimal.

## 3. Results and Discussion

### 3.1. Results of Reference GC Assays with Internal Standard Method

**Figure 4:**
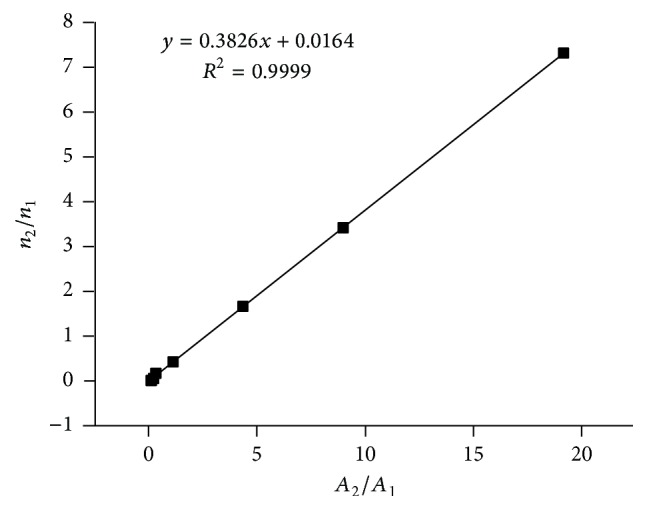
The linear relationship of internal standard method.

Based on the result of GC analytical conditions in Section 2.3, the standard compound of 2-chloropropionic acid was used to determine the relative correction factor (*f*) of ethyl 2-chloropropionate. The related data of internal standard method are listed in Table 1, which are further linearly fitted as shown in Figure 4. The linear equation is *y* = 0.3826*x* + 0.0164  (*R* ^2^ = 0.9999). As a result, *f* was determined as 0.3826 and a good linear relationship could be obtained in the range of molar ratio from 0.0362 to 7.3567.

The validation of the above GC analytical method was also studied. Standard linearity was tested using linear regression and ethyl 2-chloropropionate together with 2-chloropropionic acid showed excellent linearity with correlation coefficient greater than 0.999 in the studied range. Within-run precision was measured using RSD for six replicate standards of 2-chloropropionic acid and ethyl 2-chloropropionate. RSD values of retention time and the peak areas for 2-chloropropionic acid were within 0.16% and 1.56%. For ethyl 2-chloropropionate, the RSD values of retention time and the peak areas were within 0.37% and 1.98%. The accuracy of the GC method was validated by adding two standards to the known concentration of their samples. As a result, average recoveries of 3 replicates were between 96.81% and 101.25%. The above results showed that the analytical method was acceptable.

### 3.2. Model Development

#### 3.2.1. Spectra Band Selection

A quantitative model was established by PLSR, in which all the wavelengths could be analyzed and processed. But part of the signals in the spectrum (produced by solvent, temperature, variance of flow rate, etc.) was not related to the target compound, and the performance of the model would be affected at the same time. A reasonable wavelength can improve the prosperity of the model with a small amount of computation [15]. The comparison of different regions is shown in Table 2. According to the results, the interval of 1000–1240 nm was finally selected to establish calibration model of ethyl 2-chloropropionate content.

**Figure 5:**
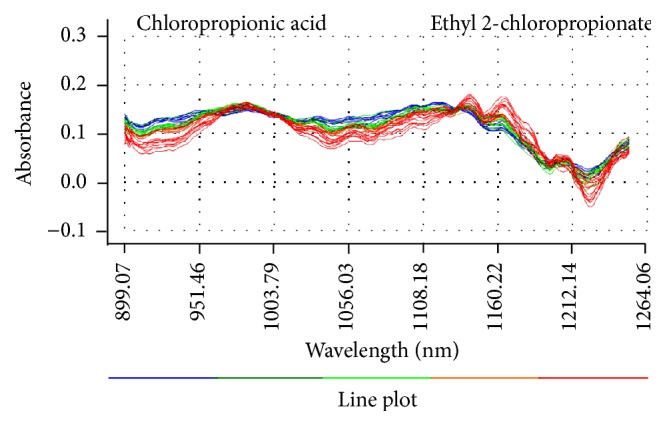
Origin NIR spectra of the synthesis process of ethyl 2-chloropropionate.

The origin spectra collected between 899.07 and 1264.06 nm in the reaction process are shown in Figure 5. It can be found that the absorbance in two regions of 1003.79–1134.40 nm and 1212.14–1240.18 nm will decrease with increasing reaction time. Because the content of 2-chloropropionic acid becomes less and less, the intensity of related absorbance band of –COOH group will decrease. However, there is a turning point appearing in 1134.40 nm, and the absorbance in the region of 1134–121.14 nm will rise continuously with the increasing content of ethyl 2-chloropropionate. The region is closely related with the characteristic absorption of –COOEt group.

#### 3.2.2. Pretreatment of Original NIR Spectra

NIR spectra of the samples, which contain much chemical information, need to be pretreated to ensure accurate analysis [16]. There are many factors that would cause interference in the spectral measurement process, so most of the NIR measurement methods require the use of chemometric treatment [17]. Taking measurement to eliminate outside interference to some extent is very necessary and helpful to optimize the performance of a quantitative model.

Table 3 shows the comparison results of related pretreatment methods and their combinations. It can be seen from the table that the various pretreatment methods can improve the performance of models to different extent. In many cases, derivatives can reduce peak overlap and eliminate linear baseline drift. But the noise level increases slightly. In addition, the standard normal variate (SNV) was applied to reduce the changes in the path length and to reclaim the light scattering. Considering that some pretreatments even exhibited negative values, these treatments were avoided. Obviously, the combination of SNV and second-order derivative method was superior to other pretreatment methods, which presented the greatest value of *R* ^2^ and the smallest RMSE. Therefore, SNV + second-order derivative was selected to pretreat the original data.

#### 3.2.3. Selection of Optimal Number of Principal Components

**Figure 6:**
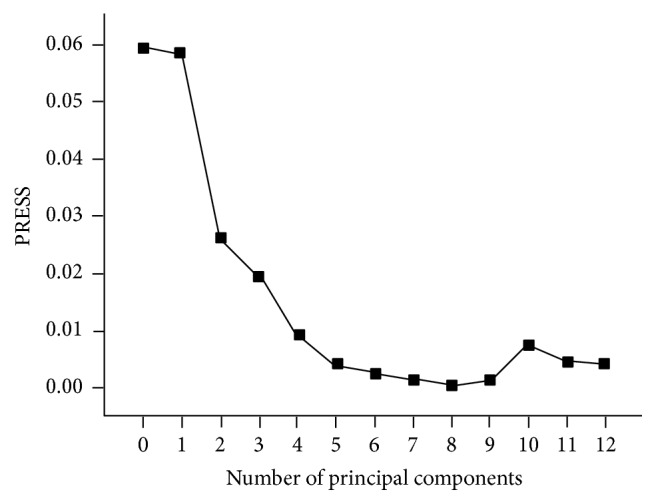
The principal PRESS distribution fraction of PLSR model.

The decomposition model of data compression with PLSR method is different from other chemical decomposition models. It explores the matrix of absorbance and the matrix of the reference value of the concentrations [18]. Respectively, scoring matrix and loading could be calculated (the two scoring matrixes could exchange alternatively). Meanwhile, this method can make the reference of concentration related to the spectrum information better. In PLSR process, cross-validation based on the samples from training set and corrected set was conducted, and the predicted residual error sum of squares (PRESS) was calculated at one time [19]. The PLSR models with 0–12 factors were investigated, and the optimum number of factors employed in PLSR was determined by PRESS. The distribution of the PRESS is shown in Figure 6. It can be concluded that the values of the previous seven main constituents are decreasing. And the result explains that the increasing main constituents are responsible for the content of ethyl 2-chloropropionate. When the principal components were less than 7, this established model was underfitting, and when the number of main components was greater than 8, the PRESS value would increase. It indicated that the number of principal components was redundant and extra factors were considered in the establishment of models. So the optimal number of principal components was determined to be 8. As comparison, the cross-validation method was also used to ascertain the number of significant factors (latent variables) in the PLS algorithm, which left out one sample at a time. The PRESS was calculated in the same manner each time a new factor was added to the PLS models, and the *F*-statistic was used to make the significance determination according to the suggestion of Haaland and Thomas [20]. The maximum number of factors used to calculate the optimum PRESS was selected as 13 and the optimum number of factors obtained by the application of PLS model was 9 as a result.

#### 3.2.4. Establishment of Regression Model by PLSR

**Figure 7:**
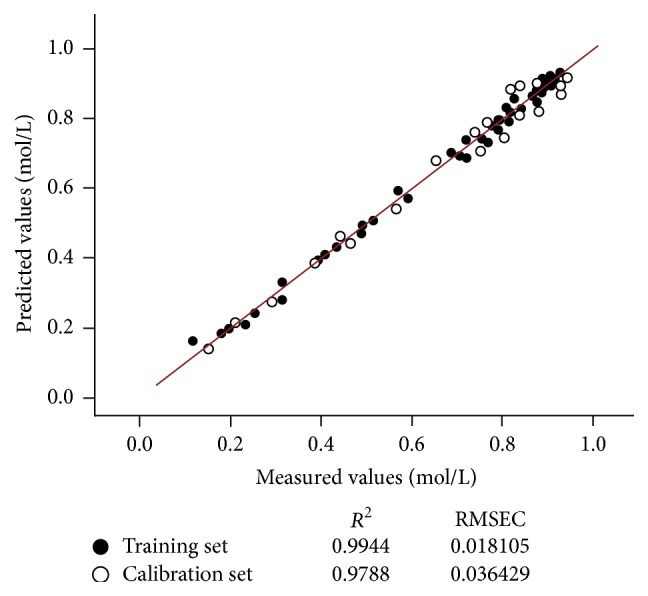
The correlation diagram of reference and NIR prediction.

Spectral data were manipulated by identifying the proper spectral region (1000–1240 nm) and choosing SNV + second-order derivative as appropriate pretreatment method, and then they were correlated with the data measured by the reference assays using PLSR method to develop calibration models [21]. After the study of optimal number of principal components, the correlation diagram of reference and NIR prediction of ethyl 2-chloropropionate in related reaction process is shown in Figure 7. The correlation coefficients (*R* ^2^) of training set and calibration set are 0.9944 and 0.988, respectively. The performance of the calibration models is assessed in terms of root mean relative standard error of prediction (RMSEP). The calibration model with the high *R* ^2^ value and the low RMSEP is considered as the ideal model.

It can be found that the predicted model and the references of ethyl 2-chloropropionate in the involved process have a similar trend. However, the uncertain factors, such as the pulse of peristaltic pump, temperature, tiny particles, and external vibration, all have impact on the NIR spectra to different extent [22]. Therefore, uncertain factors can lead to the slight difference of prediction and reference. The results of online NIR analysis and references can be also reflected by Figure 7. As a result, RMSEC of training set = 0.018105 mol/L (*R* ^2^ = 0.9944), and RMSEP = 0.036429 mol/L (*R* ^2^ = 0.9788). The above performance can indicate that the prediction model is acceptable as a method of quantitative determination.

### 3.3. Verification of the PLSR Model

On the basis of the above developed PLSR model, 21 batches of synthetic experiments have been used to verify the accuracy of the regression model. The product contents of NIR model and offline GC analysis are listed and compared in Table 4. It can be found that the difference of the two contents is small except for sample number 9, which could be the result of experimental error. After this unqualified sample is deleted, the average relative deviation can reach 1.12%, and average predicted recovery is 101.12%. The results in Table 4 can prove that the prediction ability of PLSR model is good for PAT analysis of ethyl 2-chloropropionate.

## 4. Conclusions

In the study, we investigated the synthetic process of ethyl 2-chloropropionate based on PLSR analysis and the use of NIR to achieve the online and noninvasive monitoring of the extraction process. The sampling, spectrum acquisition, and PAT system were developed and the established quantitative models can detect the dynamic change of target product content during synthetic process accurately and in real time. The origin spectrum was pretreated through first-order derivative, second-order derivative, MSG, SNV and SG smoothing, and so forth. The values of *R* ^2^ and RMSEC of PLSR quantitative calibration model indicated that it had good performance. Offline gas chromatographic analysis was used to validate the predictive ability of the model, and the results of average relative deviation and recovery rate in the validation set were ideal. In brief, NIR spectroscopy has potential to be extended to the whole reaction process, as a time-saving and continuous measuring method. And online NIR analysis technology was proved to be a fast and effective method to observe the extent of reaction successfully. As a fast, simple, and nondestructive analysis technology, online NIR detection has become a helpful tool applied in various synthetic reactions and industrial production.

## Figures and Tables

**Table 1:** The results of internal standard method.

Sample number	1	2	3	4	5	6	7
Molar ratio of ester and acid (*n*_2_/*n*_1_)	1.6658	0.4438	0.1177	0.0666	0.0362	3.4390	19.1854
Peak area ratio of ester and acid (*A*_2_/*A*_1_)	4.1061	1.1018	0.2755	0.1462	0.0792	8.9226	7.3567

**Table 2:** Results of PLSR model with different NIR regions.

Wavelength range (nm)	Slope	Offset	RMSE	*R* ^2^
Full range	0.9896	0.006853	0.02479	0.9896
900–1000	0.9795	0.013522	0.03481	0.9795
900–1240	0.9723	0.018333	0.04054	0.9723
1000–1240	0.9926	0.004884	0.02092	0.9926
1000–1100	0.9900	0.006602	0.02433	0.9900
1100–1200	0.9835	0.010941	0.03132	0.9835
1200–1300	0.9880	0.007965	0.02672	0.9880
1300–1400	0.9899	0.006665	0.02444	0.9899
1400–1500	0.9777	0.014765	0.03638	0.9777
1500–1600	0.9806	0.012825	0.03391	0.9806
1600–1700	0.9723	0.018333	0.04054	0.9723

**Table 3:** Results of PLSR model with different pretreatment methods.

Pretreatment method	Slope	Offset	RMSE	*R* ^2^
Untreated	0.9926	0.004884	0.02092	0.9926
First-order derivative	0.9885	0.007584	0.02607	0.9885
Second-order derivative	0.9935	0.004326	0.01969	0.9935
SNV	0.9910	0.005941	0.02308	0.9910
SNV + first-order derivative	0.9890	0.007271	0.02553	0.9890
SNV + second-order derivative	0.9945	0.003657	0.01811	0.9945

**Table 4:** The results of model verification.

Number	*C*_NIR_ (mol/L)	*C*_GC_ (mol/L)	Predicted recovery	Absolute deviation	Relative deviation
1	0.150	0.141	106.38%	0.009	6.38%
2	0.209	0.216	96.76%	−0.007	−3.24%
3	0.290	0.275	105.45%	0.015	5.45%
4	0.384	0.385	99.74%	−0.001	−0.26%
5	0.463	0.442	104.75%	0.021	4.75%
6	0.440	0.464	94.83%	−0.024	−5.17%
7	0.564	0.541	104.25%	0.023	4.25%
8	0.651	0.680	95.74%	−0.029	−4.26%
9	0.592	0.677	87.44%	−0.085	−12.56%
10	0.750	0.706	106.26%	0.0442	6.26%
11	0.803	0.746	107.64%	0.057	7.64%
12	0.738	0.760	97.11%	−0.022	−2.89%
13	0.764	0.788	96.95%	−0.024	−3.05%
14	0.836	0.809	103.34%	0.027	3.34%
15	0.879	0.821	107.06%	0.058	7.06%
16	0.928	0.870	106.67%	0.058	6.67%
17	0.837	0.894	93.62%	−0.057	−6.38%
18	0.816	0.883	92.41%	−0.067	−7.59%
19	0.941	0.917	102.62%	0.024	2.62%
20	0.926	0.893	103.70%	0.033	3.70%
21	0.875	0.901	97.11%	−0.026	−2.89%

Note: absolute deviation = *C*_NIR_ − *C*_GC_.

Relative deviation = (*C*_NIR_ − *C*_GC_) × 100%/*C*_GC_.

Predicted recovery = *C*_NIR_ × 100%/*C*_GC_.
